# What Effect Did the Global Financial Crisis Have Upon Youth Wellbeing? Evidence From Four Australian Cohorts

**DOI:** 10.1037/dev0000092

**Published:** 2016-02-08

**Authors:** Philip D. Parker, John Jerrim, Jake Anders

**Affiliations:** 1 Institute for Positive Psychology and Education, Australian Catholic University; 2 Institute for Education, University College London; 3 National Institute of Economic and Social Research, London, United Kingdom

**Keywords:** wellbeing, Global Financial Crisis, adolescence

## Abstract

Recent research has suggested significant negative effects of the Global Financial Crisis (GFC) on mental health and wellbeing. In this article, the authors suggest that the developmental period of late adolescence may be at particular risk of economic downturns. Harmonizing 4 longitudinal cohorts of Australian youth (*N* = 38,017), we estimate the impact of the GFC on 1 general and 11 domain specific measures of wellbeing at age 19 and 22. Significant differences in wellbeing in most life domains were found, suggesting that wellbeing is susceptible to economic shocks. Given that the GFC in Australia was relatively mild, the finding of clear negative effects across 2 ages is of international concern.

The influence of macrolevel events and conditions on psychological variables is of central interest within the social sciences (Fletcher, 2015). In particular, there is growing interest in the influence of shifts in local and global economic conditions on personality (Bianchi, 2014), mental illness (Sargent-Cox, Butterworth, & Anstey, 2011), and wellbeing (Di Tella, MacCulloch, & Oswald, 2006; Yang, 2008). Estimating the impact of such factors, however, has proven to be difficult. This is due to the use of cross-sectional designs that make it difficult to separate the influence of development (the degree to which there are changes in wellbeing that correspond to particular developmental stage) and period (cultural and economic conditions or events unique to a particular historical period; Fletcher, 2015; Schoon, 2006; Yang, 2008).

In this article, we took a multidisciplinary approach, using literature and methodological approaches from psychology, sociology, and economics to estimate the impact of the Global Financial Crisis (GFC) on the multidomain wellbeing of Australian youth. To do this we used four cohorts of the Longitudinal Study of Australian Youth (LSAY) with wellbeing measured in 12 domains. Unlike previous research, we used longitudinal data from multiple birth-cohorts to estimate the effects of a unique and pervasive economic crisis. We also used wellbeing measured across most major life domains. This is in contrast to most research to date, which has focused on a single general domain. Furthermore, we leveraged the unique opportunities afforded by the LSAY to estimate these effects at two distinct ages (19 and 22 years of age). To do this we used statistical models, rarely used in previous research, to provide counterfactual estimates of the effect of the GFC (Morgan & Winship, 2014).

## Macrocontext and Wellbeing

There has been growing interest in recent years of the effects of macrocontext (national or international conditions or events) on individual factors in psychology (Fletcher, 2015). However, the idea that dramatic changes in the global environment can have meaningful influence on individual psychology is not a new one. C. Wright Mills (1959/2000, p.3) laid the groundwork for this area of inquiry, stating “neither the life of an individual nor the history of a society can be understood without understanding both”. In a pioneering study, Glen Elder’s (1999) research on children growing up in the Great Depression prompted consideration of not only the influence of macrolevel conditions on progress and frustration in development, but also how such effects filter through to young people via links with local institutions, social ties, and family networks. Elder (1999) noted effects of the Great Depression on social wellbeing, psychological health, and hope and optimism for the future; particularly among those who were younger and thus less cognitively developed. In addition, Elder drew attention to the effect of economic downturns on populations of youth as a whole, in addition to those suffering abject and persistent deprivation (see Elder & Caspi, 1988). Thus, one needs to consider the effects of economic downturns on factors such as wellbeing across whole cohorts (Jahoda, 1988).

Recent research by Di Tella et al. (2006) found that a country’s economic position has significant effects on wellbeing. Indeed, Di Tella et al. indicated that rising unemployment that results from economic hardship has a critical effect not only on those who lose their job, but for the population as a whole. These effects were observed across a range of macroeconomic events including recessions, changes in GDP, inflation, and the relative generosity of the welfare system. Schoon (2006) considered cohorts of British people born in 1958, thus growing up in a golden age of economic stability and prosperity, and those born in 1970, thus growing up in more economic vulnerable times. Schoon reported that growing up in times of economic prosperity seems to be a protective factor against psychological distress and promotes wellbeing. Conger, Rueter, and Conger (2000), studying the effects of the severe economic downturn in the rural midwest of the United States found that economic distress affected young people’s wellbeing via its impact upon parents’ mood and parenting behavior. Finally, Forkel and Silbereisen (2001) considered the effect of the reunification of Germany on development. Using a family stress model framework, they found that economic uncertainty had an effect on child wellbeing via parents depressed mood in the West, but less so in the more significantly altered society in the East.

## The GFC and Wellbeing

In relation to the GFC, a review by Clark and Heath (2014) found dips in trends in happiness and social wellbeing, including trust and experiences of prosocial behavior in the United Kingdom and the United States. In Australia, Sargent-Cox et al. (2011) focused on the influence of the GFC on Australian seniors, suggesting that this group was at particular risk due to vulnerability in retirement savings as well as fear spread by the Australian media. They also found significant increases in depression and anxiety. Likewise the recent UNICEF Innocenti report (Fanjul, 2014) found that in 29 of the 41 OECD and non-OECD EU countries wellbeing decreased and experience of everyday stress increased from 2007 to 2013. They attributed this impact as likely due to the GFC.

Taken together, the literature to date suggests three important considerations. First, changes in macrocontexts, and economic conditions in particular, can have meaningful impacts on wellbeing. Second, these may have an impact upon everyone (i.e., those directly and indirectly affected). Third, consideration of general wellbeing should be supplemented by consideration of domain specific measures within multiple life domains, given findings that social domains of life appear to be vulnerable to economic conditions.

## Youth and Vulnerability

Although Elder (1999) focused on the effect of the Great Depression on youth, recent research has tended to focus on the elderly as a group of particular vulnerability. Although the elderly were particularly exposed to the GFC (e.g., Sargent-Cox et al., 2011), there are important reasons to also consider the developmental period ranging from the transition from high-school to the mid-20s. Here we explore the biological, social, and economic reasons for this.

Steinberg (2009, 2013) has highlighted convincing biological, behavioral, and neurological evidence to extend the definition of adolescence up to mid-20’s. Steinberg’s (2014) argument is both social, noting that youth are now becoming financially and socially independent at later ages, and biological, with evidence of continued and significant brain plasticity well into the mid-20s. Steinberg noted that this malleability means that young people are particularly vulnerable to toxic contexts that can lead to lifelong negative impacts. Cummins (2014) likewise noted that wellbeing is particularly volatile during adolescence due to heightened biosocial change. This is consistent with the work of Elder (1999) who noted that age was negatively related with impact of the Great Depression, hypothesizing that ongoing cognitive development meant that hardship had a more severe and long lasting impact.

Socially, not only is the post-high-school period defined by identity formation and uncertainty in social and occupational roles (Arnett, 2000) but it is a period in which developmental transitions are both plentiful and of considerable importance to long-term status attainment (Guo, Parker, Marsh, Morin, 2015; Parker, Lüdtke, Trautwein, & Roberts, 2012; Parker et al., 2012; Parker, Thoemmes, Duinveld, & Salmela-Aro, 2015). The life span theory of control indicates that those making the transition from formal schooling to tertiary education or the labor market are particularly at risk of contextual events and influences (Heckhausen, Wrosch, & Schulz, 2010; Heckhausen & Schulz, 1995; see also Dietrich, Parker, & Salmela-Aro, 2012). Such a period is defined by the convergence of developmental tasks from multiple life domains (educational, occupational, social, family, romantic, and values) and, as such, is one of the most critical developmental periods (Zarrett & Eccles, 2006). From the perspective of life span theory of control (Heckhausen & Schulz, 1995) the particular danger of macroeconomic events, like the GFC, would be the potential to knock youth off a typical developmental track; delaying transitions, interfering with increasing independence from parents, and extending periods of career and educational uncertainty. For example, research on transition delays provides evidence that even a relatively short delay can have ongoing consequences for status attainment well into adulthood (see Haase, Heckhausen, & Köller, 2008; Heckhausen & Tomasik, 2002; Parker et al., 2015).

Economically, not only is unemployment particularly high during this developmental period, but in Australia, the United Kingdom, and the United States the jump in unemployment levels during the GFC for those aged 16 to 24 was notably larger than for the working population as a whole; youth unemployment in Australia jumped from 8.9% to 13.8%, whereas overall unemployment grew from 4% to almost 6%, in the period of 2008 to 2011 (our calculations are based on Australian Bureau of Statistics data). As noted above, both unemployment and the risk of unemployment has a particularly detrimental effect on wellbeing (Clark, Georgellis, & Sanfey, 2001). The risk of unemployment can cause young people to make different choices about their educational and occupational plans than they otherwise would, which can put them at a distinct disadvantage when competing with their near age peers who entered this developmental period at a more economically advantageous time (see Kahn, 2010). Finally, at the post-high-school transition young people are increasing independence via entry into the labor market or tertiary education, yet they also remain strongly connected to parents (Parker, Lüdtke et al., 2012). As such, the wellbeing of young people may suffer from both their own exposure to economic downturns but also that of their parents as suggested from a family stress model perspective (Conger et al., 2000).

## Multidomain Wellbeing

Psychologists, economists, and sociologists have all been interested in the influence of both micro- and macrolevel conditions on wellbeing. A common thread across much of this research is general or aggregated wellbeing (e.g., life satisfaction). There is, in contrast, relatively little attention given to how such events might differentially affect multiple life domains. Part of the reason is that it is difficult to determine how many and which life domains to cover. As Cummins (1996) noted, if every human action is considered a life domain, true multidimensional measurement becomes impossible.

Derived from the work of Cummins and colleagues, however, youth surveys of the Australian population have covered between 12 to 14 life domains focusing on achievement, social life, community engagement, perspectives on the future, and living standards. These domains are derived from empirical research on what most participants consider to be important and have been used over long periods of time, across countries, and age groups. This provides strong evidence of validity and utility of multiple dimensional measures of wellbeing in these areas (see Cummins, 2014; Tomyn, Fuller Tyszkiewicz, & Cummins, 2013, for a review). As Cummins (2014) noted, there is value in a parsimonious multidomain approach, and the domains that are used here capture the domains that are relevant for the majority of young people (Tomyn et al., 2013).

Thus, taking a multidimensional perspective, we consider the degree to which there are differential impacts of events like the GFC on wellbeing measured in different domains. As noted above, there is some evidence to suggest that social wellbeing and optimism for the future is particularly at risk during economic hard times (Clark & Heath, 2014; Elder, 1999; Lau et al., 2008), yet research in this area has been relatively limited in the number of domains explored.

## Hypotheses

Empirical research suggests economic conditions can lead to significant changes in wellbeing. This literature, however, has tended to use cross-sectional studies without the ability to follow individuals over time. Here we make use of the unique opportunities afforded by the LSAY datasets, which follow young people from four birth cohorts for up to 10 years. The nature of the LSAY data, four birth cohorts measured roughly three years apart, allows us to compare the influence of the GFC at two distinct ages in the post high-school transition period (i.e., age 19 and 22). As can be seen in Table 1, the 19-year-old age group captures much of the movement of young people from high-school to tertiary education or the labor market. At age 22, young people appear to have mostly made this transition. The comparison of these age groups is opportunistic (i.e., due to the possibilities afforded by the data), however, and thus we have little evidence on which to assume the GFC would have differential effects. On this basis, we put forward the following hypotheses:

*Hypothesis 1:* The GFC will have a negative impact upon young people’s wellbeing across the major domains of importance to late adolescents.[Table-anchor tbl1]

*Hypothesis 2:* We expect the influence of the GFC to differ by life domain, with particular impact on domains related to social life and long-term prospects.

*Hypothesis 3:* As existing research base is not yet large enough on which to make a strong hypothesis, we do not anticipate that there will be differences in the size of the effect of the GFC at age 19 compared to 22.

## Method

### Participants

**Figure 1 fig1:**
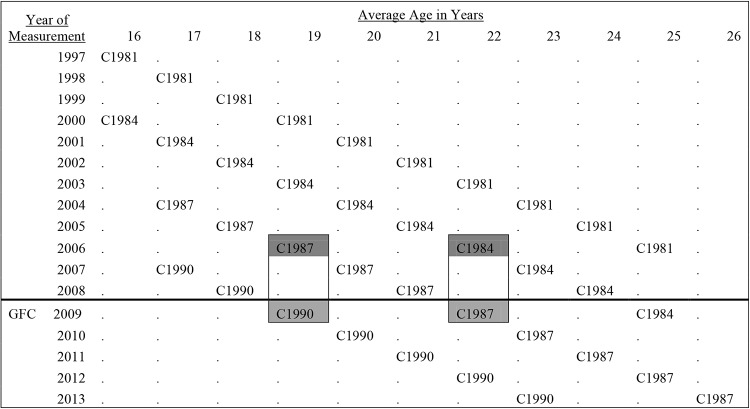
Age and year of data collection. C1981 = 1981 birth cohort; C1984 = 1984 birth cohort; C1987 = 1987 birth cohort; C1990 = 1990 birth Cohort. Light gray = the year of the Global Financial Crisis (GFC). Dark gray boxes = the critical comparison at age 19 and 22 in the propensity score matching (PSM) and difference-in-differences (DID) models.

Four cohorts of the LSAY database were used. Two of those cohorts did not go through the GFC during the time period covered in the study: birth cohorts 1981 (*n* = 9,738; ages covered 17–25) and 1984 (*n* = 9548; ages covered 17–26). Two cohorts did experience the GFC during the study: birth cohorts 1987 at age 22 (*n* = 9,378; ages covered 17–26) and 1990 at age 19 (*n* = 9,353; ages covered 17–23). The cohorts are named after the modal birth year. The structure of the data is represented in Figure 1. The 1981 and 1984 cohorts reflect representative samples of Australian year nine students with wellbeing data collected 2 years later. The 1987 and 1990 cohorts represent longitudinal extensions of the Programme for International Student Assessment (PISA), a representative sample of 15-year-olds where wellbeing data was collected a year later. Harmonization was based on modal grade in school rather than age in years. As a result there is a difference of several months in the average age of the cohorts for the waves of interest with the average age gradually increasing from 1981 to 1990 cohorts. This may be due in part to differences in how data was collected, but may also reflect a growing preference for later school intake ages by parents (see Edwards, Taylor, & Fiorini, 2011). Population weighted demographics for each cohort can be found in Table 1.[Fig-anchor fig1]

All cohorts used a two-stage sampling procedure. The primary sampling unit was schools, selected with probability proportional to size. A random sample of students was then selected from within each school. Weights are provided that aim to account for (a) particular design effect including the disproportionate sampling of schools and (b) participant attrition (Marks & Long, 2000). Thus the sample weights aim to provide unbiased estimates of the population consistently across the waves of the study.

### Materials

#### Wellbeing

Wellbeing was assessed using a measure similar to the Personal Wellbeing Index (PWI) originally developed by Cummins and colleagues (e.g., Cummins, Eckersley, Pallant, Van Vugt, & Misajon, 2003). Versions of this measure have been used in a number of large-scale panel studies in Australia and beyond, including in all LSAY cohorts. As such it provides a critical insight into historical trends in wellbeing of Australian youth. There are 12 domains covered by this instrument. Two additional domains relating to the economy and the way in which the country is being run were excluded due to not being present at critical waves of the study. All variables begin with the stem “How happy are you with [DOMAIN]” (see below for suffixes), with response scales varying from 1 (*very happy*) to 4 (*very unhappy*). To aid interpretation, these answer points were reverse scored such that higher scores reflected greater happiness. An additional response point was included representing “can’t say/don’t know.” This choice was selected by less than 1% of the sample on average and never more than 4% for any question in any wave. This response was coded as missing for the purposes of the current study. Abbreviations will be used for the 12 wellbeing variables (exact item suffix in brackets) as follows: general (your life as a whole), living (your standard of living), home (your life at home), future (your future prospects), career (your career prospects), work (the work you do, at study, at home or in a job), money (the money you get each week), leisure (what you do in your spare time), location (where you live), social (your social life), people (how you get on with people in general), and independence (your independence; being able to do what you want).

#### GFC

The GFC is generally considered to have begun during 2008. However, the impact on Australia and the individuals in the study likely came later. Sargent-Cox et al. (2011) made the case that the impact of the GFC on Australians, and particularly the psychological impact, should be dated to 2009. We thus consider the GFC to have occurred when participants were aged 19 in the 1990 cohort and 22 for the 1987 cohort. Marking the GFC at 2009 is both consistent with previous research, captures both the dramatic jump in unemployment levels that centered on this period and the zenith of media reporting on the GFC where there was a particular environment of heightened “panic, anxiety, and insecurity” (Sargent-Cox et al., 2011, p. 1105).

### Analysis

#### Age-period-cohort effects

A long running concern in developmental psychology has been how to disentangle the effects of age, period, and cohort (see Baltes & Nesselroade, 1970; Schaie & Strother, 1968). Age effects are concerned with how old an individual is, cohort effects are concerned with the shared experiences of those who grow up in a similar historical context, whereas period effects are concerned with the impact of particular events that occur at a given time in history (see Schoon, 2006; Yang, 2008). It is these period effects, and in particular changes in wellbeing that occurred after 2009 that are the focus of the current research. Such research is limited by the requirement of having multiple cohorts of data that cover at least part of the life span.

Even when such data are available, there are concerns about identifying such effects given they are confounded (e.g., age = period-cohort). To account for this we consider age as fixed (e.g., we only ever compare 19-year-olds to other 19-year-olds). Second, we aim to minimize the influence of cohort by making statistical comparisons between cohorts who were born closest in time thus ensuring that they share much of the same historical context (see Figure 1). Thus, when considering the influence of the GFC at age 19, we compare the 1990 cohort (as the exposed group) to the earlier 1987 cohort (as the nonexposed group). When considering the effect of the GFC at age 22, we compare only the 1987 cohort (as the exposed group) to the earlier 1984 cohort (as the nonexposed group; see Figure 1).

#### Counterfactual reasoning

In addition to concerns relating to isolating period effects, we were also concerned with providing estimates of the effect of the GFC that were as close to causal as the data would allow. To do this, we aimed to find counterfactual conditions that serve as an indication of what would have occurred to a variable of interest had a given event not occurred (Morgan & Winship, 2014). Put simply, in the case of the current research, we ask the question “What if the GFC never happened?” In the current research a birth cohort that experienced the GFC at a particular age serve as the exposed group (i.e., experienced the GFC at age 19 or 21) and the closest earlier cohort at the same age serves as the nonexposed group (i.e., did not experience the GFC at age 19 or 21). To increase our confidence that the control group acts as a sufficient counterfactual for the treatment group we used two approaches common in sociology and economics; namely a matching and a difference-in-differences (DID) technique.

#### Propensity score matching

Matching aims to find strategic subsamples of individuals in the exposed and nonexposed groups that either match participants across groups exactly on a small number of critical confounding variables, match approximately on a large number of confounding variables, or some combination of the two (Morgan & Winship, 2014). In the current research we used a mixture of exact and approximate matching via a propensity score matching (PSM) approach. Here participants in the exposed and nonexposed groups were matched exactly on exogenous demographic variables (gender, state of residence, social class [Erickson-Goldthorpe-Portocarero schema; Ericson, Goldthorpe, & Portocarero, 1979], and Indigenous status) and postschool pathway variables (number of years of high-school completed, labor market status [employed, unemployed, not in labor market], and tertiary education status [enrolled, completed, dropped out, not relevant] measured at age 18 for the 19-year-old comparison and 21 for the 22-year-old comparison). Participants were also propensity matched on age in days and all wellbeing variables up to the year prior to the GFC.

The aim of PSM is to create samples of exposed and nonexposed individuals who are similar (or balanced) on a wide range of potentially biasing covariates. Initial analysis consisted of modeling the relationship between the covariates and presence in either the exposed or nonexposed groups. We used logistic regression to estimate the propensity score and, based on these scores, we used nearest neighbor matching with matches allowed when participants were within .20 of the standard deviation of the logit of the propensity score. As noted above, exact matching was used for several demographic, educational, and occupational status variables. One-to-one matching was used, without replacement (see Stuart, 2010; Thoemmes & Kim, 2011, for a review). Propensity score estimation and matching were done with the MatchIt package in R (Ho, Imai, King, & Stuart, 2011) and regression with clustered standard errors for school membership was conducted with the survey package (Lumley, 2011). Hypotheses were tested using equation 1.

$$Y=\alpha +\beta COHORT+\gamma PRE\_Y+e_{i,j}$$ 1

Here γ represented the effect of the wellbeing variable PRE_Y before the GFC (age 18 for the 19 year-old comparison and 21 for the 22-year-old comparison), β is the parameter of interest—the difference in Y between the GFC exposed cohort (coded 1) and control cohort (coded 0). Subscript *j* was the school that individual *i* was in at wave 1. Importantly, PSM allowed us to match participants on both grade in school and age in days, thus ensuring participants were similar in both biological and social developmental stage at the comparison point.

#### DID

**Figure 2 fig2:**
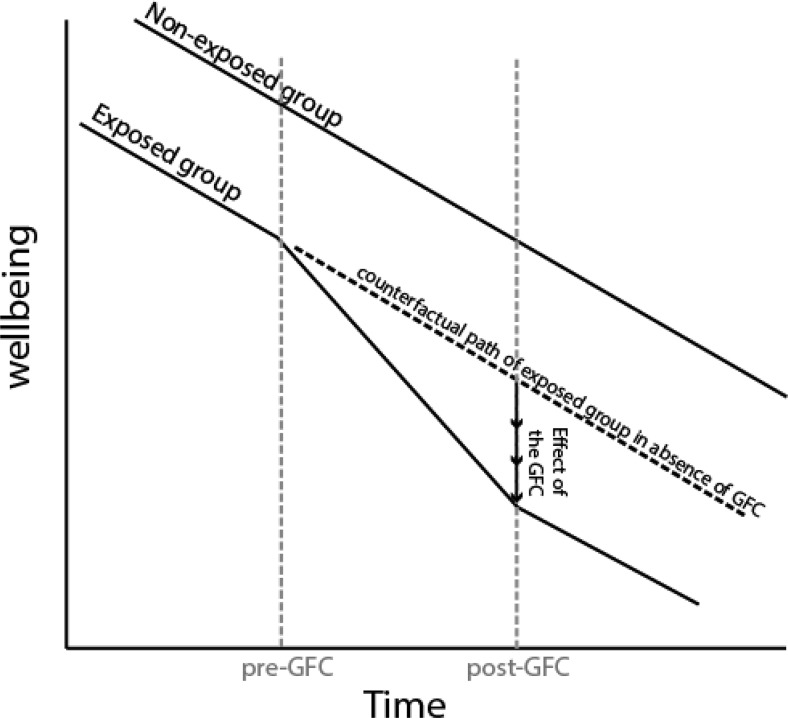
Hypothetical example of a difference-in-differences model. GFC = Global Financial Crisis.

As a robustness check, and to provide population estimates, we also adapted the logic of DID to estimate the GFC influence across cohorts. A DID approach estimates trends in a variable of interest in an exposed and nonexposed group. It assumes that both trends are essentially parallel and would remain so had an event of interest (e.g., the GFC) not occurred. A DID approach estimates the shift from parallel trends at the exposure point (see Figure 2). The assumption is that this discontinuity in parallel trends provides an estimate of the effect of exposure to a given event (Angrist & Pischke, 2014, provide a number of applied examples).[Fig-anchor fig2]

Typically this model is used to explore the potential effect of a treatment in two or more contemporaneous groups; one in which the treatment is present and one where it is not. For the GFC, however, young people either went through the historical period at a particular developmental stage or they did not. The multiple cohorts of LSAY, however, allow us to extend the logic of the DID approach to noncontemporaneous groups, given that the same measures were collected using the same survey collection procedure on participants of approximately the same age. As noted above, we thus make the assumption that cohort effects are negligible.

Following, Angrist and Pischke (2014) we fitted two sets of models. The first was a basic DID model specified in Equation 2:

$$Y=\alpha +\beta COHORT+\gamma GFC+\delta \left(COHORT\times GFC\right)+e_{t,i,j}$$ 2

Where β is the first order estimate of cohort on the wellbeing variable Y, γ is the first order estimate of the GFC and δ is the parameter of interest (i.e., whether there was a shift in trend for the GFC exposed cohort at the time of the GFC; see Figure 2). The subscripts *t* refer to individual observations at a given time wave, *i* refers to individual participants under which observations were nested, and *j* relates to the primary sampling unit which, in our case, was the school the individuals were in at the first wave of data collection.

Exploiting the fact that we had more than two waves of data, we also tested a model in which the assumption of common trends was partially relaxed. This second model was estimated using Equation 3:

$$Y=\alpha +\beta COHORT+\gamma GFC+\delta \left(COHORT\times GFC\right)+\pi TREND+\eta (TREND\times COHORT)+e_{t,i,j}$$ 3

In Equation 3, π and η are included to relax the assumption of common trends, and allow for cohort specific linear trends. All other terms remain consistent with Equation 1.

#### Missing data and survey design

As noted above the LSAY database has a complex design. To account for this a series of weights were applied to ensure estimates were representative of the Australian population. Finally, even with the use of attrition weights there remains missing data holes where participants have failed to complete a particular item within a given wave. To account for this various complexities we (a) provide clustered standard errors for individual observations nested within participants who were themselves nested within schools; (b) apply sample and attrition weights; and (c) multiply impute wave specific missing data. Imputation was achieved using a bootstrapped expectation maximization approach (Honaker, King, & Blackwell, 2011). Given that nonattrition related missing data was generally small (<5%), five imputations was considered sufficient.

## Results

### Graphical Results

**Figure 3 fig3:**
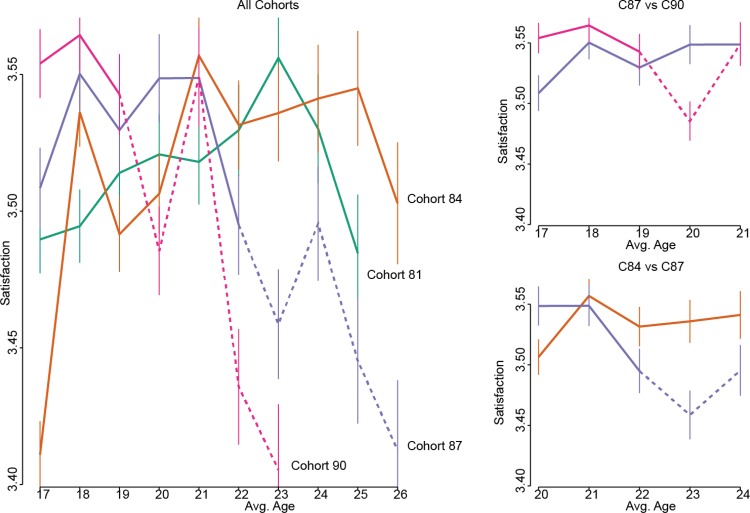
Trends in satisfaction with life in general for four cohorts. Solid lines represent observations from before the Global Financial Crisis (GFC). Dotted lines are observations after the GFC. See the online article for the color version of this figure.

The means and confidence intervals for each cohort were plotted in the following manner (see Figure 2, e.g., plot). First, all cohorts were plotted on a single graph with solid lines representing observations that occurred before the expected impact of the GFC (i.e., 2009–2010). Second, two close-up plots for each wellbeing domain were created, highlighting particular comparisons of interest. These close-up plots also provide insights into the comparisons of interest for the PSM and DID models. The first close-up compares the 1990 and 1987 cohorts at ages 17 to 21. The second compares the 1990 and 1984 cohorts at ages 20 to 24. Given space restrictions, we provide an example plot for general wellbeing only (see Figure 3). However, all graphs, means and 95% confidence intervals, and an interactive graph are available from the paper website at https://pdparker.github.io/GFCweb/. Microdata is available by application from the Australian Data Archive (https://www.ada.edu.au/).[Fig-anchor fig3]

A visual inspection of all the graphs suggested that the 1987 and 1990 cohorts had similar (or slightly higher) levels of wellbeing across domains than the earlier cohorts before the GFC. However, a relatively large gap emerges between the earlier and later cohorts, starting at age 19 for the 1990 cohort and age 22 for the 1987 cohort. Thus, results were consistent with the hypothesis that the GFC had a negative impact on wellbeing. Interestingly, there was some evidence of recovery in 2011 (ages 21 for the 1987 cohort and 24 for the 1990 cohort), where in many cases the wellbeing levels returned to those of the other cohorts before again diverging and growing progressively larger. Finally, the first wave of the 1984 cohort was well below trend and may represent an outlier for consideration in later models.

The close-up graphs provide strong evidence for the negative impact of the GFC with most of the relevant contrasts displaying overlapping confidence intervals in the years prior to the GFC before diverging. It was on this basis that we explored the hypotheses using PSM and DID models.

### PSM

Two sets of PSM models were estimated; one comparing the 1990 with the 1987 cohort at age 19 and one comparing the 1987 to the 1984 cohort at age 22. Matching was done exactly on gender, social class, state of residence, and Indigenous status; as well as labor market status, university status, and number of years of high-school completed. Propensity matching was done on age in days and all pre-GFC wellbeing variables. Negative effects indicate that the GFC exposed cohort was lower on wellbeing than the comparison cohort (see Table 2 for results).[Table-anchor tbl2]

Matching suggested that the 1990 and 1987 cohorts were very similar with only 3% of the 1,365 assessed terms indicating a prematching difference of greater .20 of a standard deviation. After matching no term displayed a difference of greater than .12 standard deviation units. Prematching the sample size was 12,390. After matching this was only reduced to a balanced sample of 7,604. Table 2 displays the differences in wellbeing at age 19 for the 1990 and 1987 cohort controlling for pre-GFC levels. Unsurprisingly, given the similarity between the two groups, matched and unmatched results were similar. In particular, the only factor that GFC exposure did not predict was satisfaction with money. Furthermore, nine of the 12 wellbeing domains had Cohen’s *d* differences greater than .10. In order of effect-sizes these were social life, independence, general, living standards, career prospects, leisure time, future prospects, and home life.

Matching for the 1987 and 1984 cohorts revealed a greater prematching difference with 1% of the 3,402 assessed terms displaying a Cohen’s *d* differences of .20 or greater and 7% of terms greater than .25. After matching, no term had a Cohen’s *d* difference greater than .16. This matching resulted in a decline in sample size from 9,632 cases to a balanced sample of 5,572.

Matching did result in a decline in the size of effects and the number that were statistically significant. However, eight out of 12 wellbeing factors remained significant, and of those only three had effects sizes greater than .10; namely career prospects, home life, and people in general (in order of effect size). Importantly, however, these results tended to be smaller than the comparison at age 19 but generally not significantly so. Indeed, *z* tests suggested only satisfaction with living standards, independence, and social life had significantly larger effects at age 19 that 22.

### DID Results

As a robustness check to the PSM results we ran a series of DID models. In this case two sets of models were estimated. First, we compared the 1990 cohort (who went through the GFC at age 19) to the 1987 cohort. Second, we compared the 1987 cohort (who experienced the GFC at age 22) with the 1984 cohort. Negative DID estimates represent the disadvantage of the GFC exposed cohort over the comparison cohorts in terms of wellbeing.

For the DID at age 19, we found significant results for 11 wellbeing variables when we assumed a common trend (satisfaction with money was not significant) and all 12 were significant when we controlled for cohort specific trends. Of these, nine had effect sizes larger that .10. In order of effect size these were satisfaction with leisure time, social life, future prospects, independence, work, career prospects, home life, general, and people in general. Interestingly, the GFC appeared to have a small positive effect on satisfaction with money.

At age 22, results not controlling for trend were significant in all domains but only two domains when controlling for cohort specific trends (satisfaction with career prospects and work; see Table 3). As we noted above, the first time point for the 1984 cohort was considerably off trend and thus likely exerted considerable leverage on the linear trends. Thus, we also estimated these models excluding the first wave. This resulted in seven out of 12 significant results, with only career prospects having an effect size of the GFC greater than .10. Using *z* tests, the GFC had significantly larger effects for 19-year-olds than 22-year-olds in terms of satisfaction with leisure time, where you live, social life, living standards, and future prospects (ordered in terms of size of difference).[Table-anchor tbl3]

## Discussion

impact of the GFC on multidimensional wellbeing by taking advantage of the unique opportunities provided by the LSAY data. We were able to overcome limitations in previous research via the use of multiple cohorts of longitudinal data to explore the influence of the GFC at two different ages in one general and 11 domain specific measures of wellbeing. Exploration of graphed means suggested significant divergence in wellbeing for the GFC cohorts in year 2009 to 2010. Of most concern, while there was evidence of recovery in 2011 in both the 1990 and 1987 cohorts, this gap reopened and grew larger. Using the logic of PSM and DID models, these findings were also examined statistically. There was consistent evidence of a negative impact of the GFC in most domains at age 19 with the exception of satisfaction with money; which was generally not significant and occasionally positive. The effect at age 22 was more ambiguous, though generally suggested significant effects for over half the wellbeing domains. Taken together, these results suggest significant though small differences at age 22 than at age 19 for wellbeing in at least three life domains.

### Did the GFC Significantly Affect Wellbeing?

The current research across multiple models, using multiple comparisons, and across multiple domains suggested that the GFC did have a significant negative impact on the wellbeing of young people in Australia. Such a result is important, as the GFC had a much milder influence in Australia than it did elsewhere. Indeed, although youth unemployment jumped from 8.9% to 13.8% during the GFC in Australia, it rose from under 10% to almost 18% in the United States (our calculation) during the same period. Thus, although research in other countries is needed, it is likely that the results in countries such as the United States and the United Kingdom, let alone Greece, Italy, Ireland, and Spain, was considerable. Importantly, given our focus on the population as a whole, unmoderated by individual exposure, the effect sizes of above .10 standard deviation units, and often above .15, were concerning given effects of unemployment of .50 (Lucas, Clark, Georgellis, & Diener, 2004). This suggests that for particularly vulnerable groups, for example those who experienced the largest relative loss in status or income or became unemployed, the findings may have been considerably more dramatic.

### Did Wellbeing Recover?

**Figure 4 fig4:**
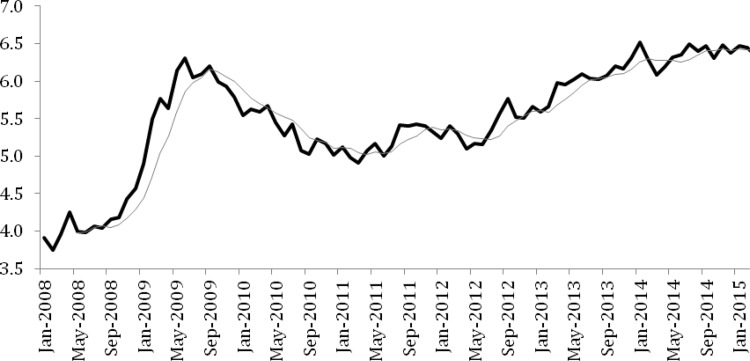
Australian unemployment rates from 2008 to 2015 based on Australian Bureau of Statistics data. Black line represents monthly unemployment. Gray line represents moving average trend line.

An interesting effect present in the trend plots for wellbeing was a drop in wellbeing in 2009, consistent with our hypothesis, before a recovering during 2010 and then a step decline again from 2011 to 2013. Although we did not provide a hypothesis for this pattern, exploration of the unemployment rates provides a potential explanation (Di Tella et al. (2006). In particular the pattern of decline and recovery is consistent across both wellbeing and unemployment. Namely, unemployment rose sharply from 2008 to 2009 before recovering just as rapidly. From 2011 unemployment then increased steadily to levels worse than those at the initial impact of the GFC (see Figure 4). Although it would be naïve to suggest that wellbeing naturally follows unemployment rates, it is fair to suggest that they do provide a proxy for general economic conditions in a country over a given time period.[Fig-anchor fig4]

### The GFC and Multiple Life Domains

Relatively little research has considered the differential effects of macro or micro contextual events on multiple domains of wellbeing. When such a comparison is made it is often done in relatively few domains. Our research was one of the few to comprehensively test the impact of events like the GFC across a wide spectrum of youth’s lives. Previous research has suggested that social domains are particularly at risk. There was some evidence that this was the case with effects on satisfaction with social life, at age 19, and getting along with people in general at age 22 being particularly affected.

For both age groups, social domains, general life satisfaction, and satisfaction with career or future prospects appeared to be most strongly affected. Such results are consistent with the developmental challenges these two groups face. In particular, these transition periods are primarily focused on the developmental tasks of developing new friendship networks and renegotiating existing relationships (Tanner, 2006). Likewise, making appropriate transitions into higher education or the labor market are crucial during these age periods (Dietrich et al., 2012). Importantly, these findings are also consistent with previous research on the GFC and the Great Depression where wellbeing in social domains and optimism for the future were particularly at risk (Elder, 1999; Clark & Heath, 2014).

Importantly, the findings suggest that the GFC had a significant impact across most life domains indicating that this event touched most aspects of young people’s lives. Importantly, the finding of small, nonsignificant results of the GFC on satisfaction with money suggests that results across domains were not merely a poisoned well effect (i.e., negative effects from one domain flooding through to all other domains). As such these findings indicate that economic hard times have a pervasive negative effect on the wellbeing of young people.

### Differential Effects of the GFC by Age Group?

Although the type of domain effects across 19- and 22-year-olds were similar, a consistent finding was that effects were routinely smaller in the older age group. This difference, however, was only consistently significant in three cases; social life, independence, and living standards. These particular domains may be associated with the many upheavals that occur during the post high-school transition (see Dietrich et al., 2012). As can be inferred from Table 1, the GFC hit 19-year-olds at the end of high-school and in a period where most of the sample was establishing themselves in either university or the labor market. Restructuring of old relationships and forming of new friendship circles after high-school is common during this period (see Tanner, 2006), which may explain why satisfaction with social life was affected more for 19-year-olds. Likewise, during this transition young people are expected to considerably increase their independence from parents (Parker, Lüdtke et al., 2012). Although not the focus of this study, it may be that the GFC meant that 19-year-olds had less financial independence and were thus less able to establish greater independence either within the family home or by moving out. The older 22-year-olds transitioned from high-school some 3 years earlier and were thus able to at least begin the developmental tasks associated with restructure old and establish new relationships and gaining independence from parents during a more prosperous period.

### Impact of Government Policy

Di Tella et al. (2006) suggested that a payment of $330 U.S. ($448 U.S. in 2009 dollars; all conversions done using Williamson, 2015) to the population in general may be sufficient to offset the effects of an economic recession on wellbeing. They do note, however, such a payment may not be sufficient for dramatic changes to economic conditions. The Australian context provides a means of exploring this hypothesis given that the government provided payments of up to $900 AUD ($597 U.S. in 2009 dollars) to 80% of the working age population and 90% of families (Hyslop, 2014). Although not the main focus of the current research, satisfaction with money was the one domain to be largely unaffected by the GFC, suggesting a positive effect of the payment may have occurred. However, any potential effect of this payment appeared to be constrained to this domain only.

### Limitations and Future Directions

There is some tension between the degree to which macroforces represent shared or qualitatively different experiences for different sectors of the community (Elder, 1999). Here we focused on the population as a whole. Although most research in psychology does focus on average treatment effects, exploring effects within particular strata is an important line for future research. This was difficult in the current case, however, where we had no data on individual exposure to the GFC, which would likely be the strongest moderator of any GFC effect (e.g., Sargent-Cox et al., 2011). Importantly, although we used rigorous designs by borrowing from the logic of DID and PSM regression in our research, the extent to which they represent causal effects is dependent on the degree to which the comparison cohorts represent true counterfactual counterparts to the GFC exposed cohorts. As we noted above we make the assumption that cohort effects are negligible. Although we aimed to design our models as close to *ceteris paribus* comparisons as possible, readers should consider the potential biasing effect of birth cohort differences. Finally, it should be noted that we used single-item measures for wellbeing in each life domain. Multi-item measures would have allowed for latent variable modeling and thus a control for measurement error.

## Conclusion

The current article was concerned with whether the GFC had an effect on young people’s wellbeing across multiple life domains. We focused on an age group that was undergoing a large number of developmental tasks at a critical period of life that has implications across the life span (Dietrich et al., 2012). We found that all domains were significantly affected in at least one case, with effect sizes often above .10 for those who were aged 19 during the GFC. Given that we were focused on a country in which the impact of the GFC was less sever than in the European Union or the United States, these effects are of international concern. As Conger et al. (2000) suggested, we cannot typically predict large-scale changes like the GFC; however, a better understanding of how such events impact young people is critical for marshaling an appropriate response.

## Figures and Tables

**Table 1 tbl1:** Demographic Data by Cohort

Demographic	Birth cohort							
	1981		1984		1987		1990	
Age (*SE*)	16.46 (.02)		16.58 (.02)		17.14 (.01)		17.35 (.01)	
Male %	48.88		51.35		50.85		48.86	
Indigenous %	2.93		3.37		2.08		2.93	
Social class								
Salariat %	62.23		48.07		74.74		73.81	
Intermediate %	28.34		34.28		12.58		12.68	
Working class %	9.42		17.65		12.68		13.51	
At age	18	21	18	21	18	21	18	21
High school years completed								
Year 12%	.27	78.70	1.08	79.41	19.74	83.19	19.64	83.18
Year 11%	86.28	9.93	86.92	9.94	64.42	8.06	63.90	8.01
Year 10%	12.21	10.15	11.02	9.65	14.99	7.96	16.01	8.42
Year 9%	1.24	1.22	.98	.99	.86	.79	.44	.39
Labor market								
Employed %	53.46	80.04	56.73	82.47	62.05	82.63	64.94	79.48
Unemployed %	11.42	9.01	10.89	7.70	12.04	6.92	8.75	8.34
Not in labor market %	35.12	10.95	32.77	9.83	25.90	10.46	26.30	12.17
University status								
Currently studying %	.01	32.94	.05	33.87	7.00	38.62	7.06	40.93
Completed %	.00	4.07	.00	6.66	.00	3.90	.03	4.55
Droped-out %	.00	6.93	.00	9.84	.49	6.56	.42	7.39
Not in university %	99.99	56.06	99.94	49.63	92.50	50.92	92.49	47.14
State of residence								
ACT %	1.96		1.93		1.89		2.03	
NSW %	33.47		32.78		31.75		32.62	
VIC %	24.32		23.45		24.14		23.96	
QLD %	18.36		20.07		19.05		19.63	
SA %	7.59		7.61		8.99		8.07	
WA %	10.57		10.55		11.18		10.23	
TAS %	2.92		2.75		2.24		2.63	
NT %	.81		.86		.75		.83	
*Note.* Age is the average age at the first wave in the analysis. Social class based on the Erickson-Goldthorpe-Portocarero schema. Three letter codes used for Australian States. All figures use population weights.								

**Table 2 tbl2:** Propensity Score Matching Results Comparing 1990 and 1987 Cohort (Age 19) and the 1987 and 1984 Cohort (Age 22)

Wellbeing	Age 19		Age 22		Difference in Postmatching age effects
	Prematching	Postmatching	Prematching	Postmatching	
General	−.157 (−.188, −.125)*	−.161 (−.213, −.110)*	−.112 (−.146, −.079)*	−.097 (−.158, −.036)*	−.064 (−.132, .004)
Work	−.084 (−.118, −.049)*	−.077 (−.140, −.013)*	−.088 (−.128, .049)*	−.066 (−.142, .009)	−.011 (−.105, .083)
Living standards	−.144 (−.181, −.107)*	−.140 (−.188, −.093)*	−.023 (−.061, .015)	−.049 (−.115, .017)	−.091 (−.171, −.011)*
Money	−.010 (−.043, .024)	−.020 (−.065, .025)	−.048 (−.087, −.009)*	−.070 (−.139, .000)	.050 (−.030, .130)
People in General	−.136 (−.171, −.102)*	−.123 (−.169, −.078)*	−.124 (−.160, −.088)*	−.107 (−.156, −.057)*	−.016 (−.084, .052)
Social life	−.155 (.187, −.123)*	−.165 (−.213, −.117)*	−.092 (−.129, −.055)*	−.081 (−.130, −.032)*	−.084 (−.153, −.015)*
Home life	−.108 (−.142, −.074)*	−.106 (−.152, −.061)*	−.080 (−.117, −.043)*	−.109 (−.165, −.054)*	.003 (−.069, .075)
Career prospects	−.142 (−.176, −.108)*	−.135 (−.183, −.087)*	−.098 (−.136, −.060)*	−.129 (−.185, −.073)*	−.006 (−.080, .068)
Future Prospects	−.103 (−.137, −.069)*	−.116 (.160, −.072)*	−.065 (−.102, −.028)*	−.052 (−.116, .011)	−.064 (−.140, .012)
Independence	−.155 (−.189, −.121)*	−.165 (−.219, −.112)*	−.080 (−.118, −.041)*	−.076 (−.153, .001)	−.089 (−.179, −.000)*
Leisure time	−.138 (.172, −.105)*	−.130 (−.182, −.078)*	−.067 (−.106, −.028)*	−.064 (−.129, .000)	−.066 (−.148, .016)
Where you live	−.088 (−.122, −.054)*	−.081 (−.128, −.034)*	−.040 (−.079, .000)	−.067 (−.124, −.009)*	−.014 (−.089, .061)
*Note.* Estimates are in standard deviation units of the wellbeing variable of interest. 95% confidence intervals are in parentheses. All parameters of interest can be found in the Supplementary Material.					
* *p* < .05.					

**Table 3 tbl3:** Standardized DID Estimates Comparing 1990 and 1987 Cohort (Age 19) and the 1987 and 1984 Cohort (Age 22)

Wellbeing	Age 19		Age 22			Difference in age effects
	Constant trend	Cohort specific trend	Constant trend	Cohort specific trend	Cohort specific trend: Wave 2–8	
General	−.167 (−.206, −.129)*	−.103 (−.165, −.041)*	−.205 (−.251, −.159)*	−.052 (−.106, .002)	−.093 (−.149, −.037)*	−.010 (−.095, .075)
Work	−.060 (−.103, −.017)*	−117 (−.180, −.055)*	−.145 (−.198, −.093)*	−.075 (−.133, −.017)*	−.071 (−.132, −.011)*	−.046 (−.135, .043)
Living standards	−.139 (−.192, −.085)*	−.087 (−.148, −.026)*	−.120 (−.170, −.069)*	.012 (−.049, .073)	.012 (−.053, .077)	−.099 (−.190, −.008)*
Money	−.010 (−.053, .033)	−.095 (.023, .167)*	−.128 (−.178, −.079)*	−.053 (−.109, .003)	−.034 (−.095, .027)	−.061 (−.158, .036)
People in general	−.108 (−.147, −.069)*	−.100 (−.159, −.041)*	−.171 (−.217, −.125)*	−.056 (−.111, .000)	−.079 (−.138, −.021)*	−.021 (−.106, .064)
Social life	−.148 (−.188, −.108)*	−.170 (−.230, −.111)*	−.196 (−.249, −.144)*	−.045 (−.099, .010)	−.070 (−.126, −.015)*	−.100 (−.182, −.018)*
Home life	−.098 (−.141, −.055)*	−.103 (−.162, −.043)*	−.156 (−.198, −.114)*	−.039 (−.090, .013)	−.057 (−.111, −.002)*	−.046 (−.128, .036)
Career prospects	−.123 (−.166, −.079)*	−.107 (−.169, −.044)*	−.204 (−.255, −.152)*	−.151 (−.207, −.096)*	−.157 (−.214, −.100)*	.050 (−.036, .136)
Future prospects	−.110 (−.154, −.065)*	−.143 (−.212, −.075)*	−.140 (−.188, −.091)*	−.049 (−.103, .005)	−.078 (−.131, −.024)*	−.065 (−.153, .023)
Independence	−.116 (−.156, −.077)*	−.130 (−.192, −.068)*	−.160 (−.209, −.110)*	.002 (−.056, .060)	−.042 (−.103, .020)	−.088 (−.176, −.000)*
Leisure time	−.133 (−.173, −.092)*	−.193 (−.254, −.132)*	−.136 (−.187, −.086)*	.008 (−.045, .062)	−.035 (−.092, .023)	−.158 (−.243, −.073)*
Where you live	−.055 (−.096, −.013)*	−.074 (−.133, −.014)*	−.106 (−.156, −.055)*	.040 (−.014, .095)	.049 (−.007, .106)	−.123 (−.206, −.040)*
*Note.* Estimates give the difference in differences estimate in standard deviation units of the wellbeing variable of interest. 95% confidence intervals are in parentheses and all parameters of interest can be found in the Supplementary Material.						
* *p* < .05.
